# Quality of evidence supporting the role of Chinese herbal medicine for the treatment of poststroke depression: A protocol for systematic review and meta-analysis

**DOI:** 10.1097/MD.0000000000028707

**Published:** 2022-01-28

**Authors:** Hongshuo Shi, Cengda Dong, Wenbin Liu, Min Peng, Guomin Si, Fengshan Sun

**Affiliations:** a College of Traditional Chinese Medicine, Shandong University of Traditional Chinese Medicine, Jinan, China; b First Clinical Medical College, Shandong University of Traditional Chinese Medicine, Jinan, China; c The Second Affiliated Hospital of Shandong University of Traditional Chinese Medicine, Jinan, China; d Department of Traditional Chinese Medicine, Provincial Hospital Affiliated to Shandong First Medical University, Jinan, China; e Department of Encephalopathy, Jinan Hospital of Traditional Chinese Medicine, Jinan, China.

**Keywords:** Chinese herbal medicine, meta-analysis, poststroke depression, systematic reviews

## Abstract

**Background:**

Poststroke depression (PSD) is a syndrome that occurs after stroke. The efficacy of Chinese herbal medicine (CHM) for PSD has also received widespread attention, but there is still a lack of clinical evidence because this overview evaluates the published meta-analyses (MAs)/Systematic reviews (SRs). To provide evidence for the clinical application of CHM in the treatment of PSD

**Methods:**

Two researchers searched 7 databases for SRs/MAs which are about randomized controlled trials on CHM for PSD. Two investigators use the systematic review assessment tool (AMSTAR-2), the risk of bias in systematic scale, the list of preferred reporting items for systematic reviews and meta-analysis, and the classification of recommended assessments for evaluation, development and evaluation system to assess the included SRs/MAs.

**Results:**

Our findings will be published in peer-reviewed journals.

**Conclusion:**

This study provides evidence-based medical evidence for the impact of CHM on PSD.

**Registration number:**

INPLASY202210001

## 1 Introduction

Depression is a mental disorder that seriously affects public health. There are about 322 million depression patients in the world, accounting for 6.2% of the total disease burden.^[1]^ Continuous depressed mood, accompanied by symptoms such as loss of interest or pleasure, psychomotor retardation or agitation, and loss of energy or fatigue is the main manifestation of depression, and even suicide and self-harm in serious cases.^[2]^ Poststroke depression (PSD) is a syndrome that occurs after stroke and presents with a series of depressive mental and physical symptoms, which is one of the common complications of clinical cerebrovascular diseases and directly affects the rehabilitation of stroke patients. The incidence of PSD is about 18.7% to 37.7%, accounting for about 20% to 79% of patients with ischemic stroke.^[3]^ A recent case-control study indicates that PSD increases disability by more than 15% in ischemic stroke survivors.^[4]^

Antidepressants are currently recognized as the main treatment for PSD. The most commonly used antidepressants are tricyclic antidepressants (TCAs), monoamine oxidase inhibitors (MAOIs), selective serotonin reuptake inhibitor (SSRIs), serotonin and noradrenaline reuptake inhibitors (SNRIs) and noradrenergic and specific serotonergic antidepressants (NaSSAs).^[5,6]^ These chemicals are effective in controlling depression-related symptoms, however, the use of antidepressants in PSD could increase the risk of certain adverse events including tremor, sexual dysfunction, urinary retention, blurry vision, hypotension, and severe insomnia.^[7]^ Besides, the use of antidepressants also carries the risk of drug-drug interactions because most patients concurrently take other types of drugs.^[8]^ Cognitive behavioral therapy (CBT) is regarded as one of the most effective psychotherapies and non-pharmacological interventions for depression, however not all people will benefit from CBT, and some people may respond better to other interventions than CBT.^[9]^ Therefore, due to the limitations of currently available PSD treatments, alternative and complementary medicines come into prominence.

Numerous PSD patients are contraindicated to antidepressants, so it is of great clinical value to develop an effective and safe PSD replacement therapy to supplement and replace existing antidepressant-centered treatment strategies. As one of the modalities of complementary and alternative medicine, Chinese herbal medicine (CHM) has a certain therapeutic effect on PSD because of its multi-target multi-compound nature that potentially benefits neurological function, rehabilitation outcome, quality of life, and depressive symptoms.^[10]^ Meta-analyses (MAs)/Systematic reviews (SRs) are thought to be the reliable criteria for evaluating the effectiveness of therapeutic interventions, but their methods must strictly adhere to a set of guidelines to minimize the bias in answering specific research questions.^[11]^ Nevertheless, a large proportion of SRs/MAs authors do not strictly adhere to the criteria above, which may lead to low-quality reviews and difficulty in providing convincing results and conclusions. We obtained a number of published systematic reviews (SRs)/meta-analyses (MAs) that reported the effectiveness of CHM on PSD by searching several necessary databases, but their methodological and quality of evidence has not been evaluated. Therefore, we designed and composed an overview to summarize the evidence on the safety and effectiveness of CHM for PSD.

## 2 Methods

### 2.1 Research registration

We have registered our protocol on the INPLASY website with the number INPLASY202210001 (https://inplasy.com/). This overview of systematic reviews was conducted strictly in accordance with the Preferred Reporting Items for Overviews of Systematic Reviews including the harms checklist (PRIO-harms).^[12]^ Our research is secondary research based on clinical research. Therefore, no ethical approval is required.

### 2.2 Inclusion and exclusion criteria

The inclusion criteria were as follows: (a) study design: SRs/MAs based on randomized controlled trials; (b) participants: the participants had PSD diagnosed according to any authoritative diagnostic criteria, no restrictions on sex, age, race, onset time, or the source of cases; (c) intervention: CHM therapy (The forms of CHM including purification for injection, decoction, patent drug, preparation, et al) versus conventional PSD drugs or CHM therapy combined with conventional PSD drugs versus conventional PSD drugs alone; and (d) outcomes: effective rate, Hamilton Depression Rating Scale, NIH Stroke Scale, Barthel Index, Scandinavian Stroke Scale, Treatment Emergent Symptom Scale, Neurological Function Defect Scale, the severity of neurological impairment scores and potential gastrointestinal and neurological adverse events.

The exclusion criteria were as follows: (a) animal studies; (b) overviews, network MAs, and narrative reviews; (c) studies in which the required data were unavailable; (d) Conference abstract.

### 2.3 Search strategy

Two independent researchers conducted a literature search. Literature searches were conducted in the Cochrane Library, PubMed, Web of Science, EMBASE, China National Knowledge Infrastructure (CNKI), Wanfang Database, SinoMed, Chongqing VIP, and from their inceptions to November 25, 2021. The search query consists of a combination of keywords and free words. We searched the above databases through the following key terms: Chinese herbal medicine, traditional Chinese medicine, stroke, depression, systematic reviews, and meta-analysis. We also manually searched the references of related articles. The specific search strategy is modified according to different databases. Table 1 provides the search strategy for the PubMed database.

**Table 1:** Search strategy of PubMed.

#1	“Stroke”[Mesh] OR “Cerebrovascular Accident”[Title/Abstract] OR “Brain Vascular Accident”[Title/Abstract] OR “Stroke, Cerebrovascular”[Title/Abstract] OR “Cerebrovascular Apoplexy”[Title/Abstract] OR “Acute Stroke”[Title/Abstract] OR “Cerebrovascular Accident, Acute”[Title/Abstract]
#2	“Depression”[Mesh] OR “Depressive Symptom”[Title/Abstract] OR “Emotional Depression”[Title/Abstract]
#3	#1 AND #2
#4	“Chinese herbal medicine”[Title/Abstract] OR “herbal medicine”[Title/Abstract] OR “traditional Chinese medicine”[Title/Abstract] OR “TCM”[Title/Abstract] OR “herbals”[Title/Abstract] OR “CHM”[Title/Abstract] OR “Chinese Medicine”[Title/Abstract]
#5	“systematic review”[Title/Abstract] OR “meta-analysis”[Title/Abstract] OR “meta-analysis”[Title/Abstract] OR “systematic review”[PT] OR “Systematic Reviews as Topic”[Mesh] OR “meta-analysis”[pt] OR “Meta-Analysis as Topic”[Mesh]
#6	#3 AND #4 AND #5

### 2.4 Eligibility assessment and data extraction

**Figure 1:**
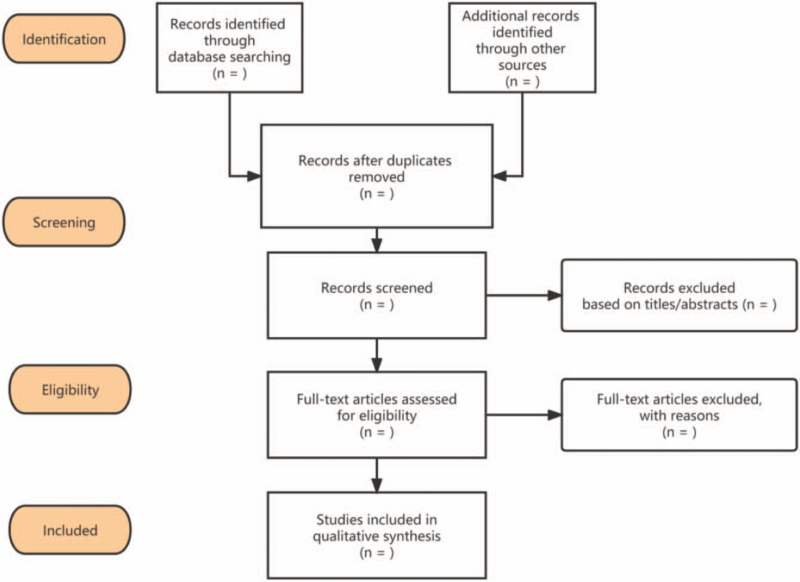
The flowchart of the screening process.

Two independent researchers independently screened the retrieved documents, first deleted duplicate publications, read the title abstract, and finally read the full text to determine its eligibility (Fig. 1). Any disagreements in the evaluation process will be resolved by the third reviewer through negotiation and arbitration. The following data were extracted by 2 independent researchers: first author, country, year of publication, number of included trials and participants, interventions, results, quality assessment methods, and the main conclusions of each included review.

### 2.5 SRs/MAs quality assessment

#### 2.5.1 Assessment of methodological quality

The Assessment System for Evaluating Methodological Quality 2 (AMSTAR-2)^[13]^ scale was used to assess the methodological quality of the included SRs/MAs. It consists of 16 items, 7 of which are critical areas (2,4, 7, 9, 11, 13, and 15). Each item is assessed using 3 assessment options, yes, partially yes, or no.

#### 2.5.2 Assessment of risk of bias

The risk of bias of the included SRs/MAs is assessed by the risk of bias in systematic^[14]^ scale. The scale was completed in 3 stages to assess the overall risk of bias. The results are judged as “low,” “unclear,” or “high.”

#### 2.5.3 Assessment of reporting quality

The list of The Preferred Reporting Items for Systematic Reviews and Meta-Analyses^[15]^ was used to assess the quality of each SR/MA report based on the following areas: (a) title, (b) summary, (c) introduction, (d) method, (e) result, (f) Discussion, (g) funding. It consists of 27 projects, with a focus on reporting methods and results in a meta-analysis. Based on the completeness of the project information report, each project is considered “yes” (full report), “partial yes” (partial report) or “no” (no report).

#### 2.5.4 Assessment of quality of evidence

The Grading of Recommendations, Assessment, Development and Evaluation system^[16]^ was used to assess the quality of the evidence of the included SRs/MAs, downgrading from 5 aspects: research limitations, inconsistencies, indirectness, imprecision, and publication bias.

### 2.6 Data synthesis and presentation

In this overview, an objective description was used. The characteristics and results of each SR/MA and the evaluation results of Assessing the Methodological Quality of Systematic Reviews 2, Risk of Bias in Systematic reviews, Preferred Reporting Items for Systematic Reviews and Meta-Analyses, and Grading of Recommendations, Assessment, Development and Evaluation are reported in the form of a list.

## 3 Discussion

Depression is a common complication after stroke and tends to interfere with daily activities, functional recovery and rehabilitation. As a widely used alternative treatment for poststroke rehabilitation, CHM has attracted researchers’ attention for its efficacy in alleviating depressive symptoms in patients with stroke. There have been several relevant clinical trials of SRs/MAs to explore the efficacy of CHM for PSD, and on this basis we conducted a comprehensive evaluation of SRs/MAs of different quality, further explored the reliability of the results, and provided a theoretical basis for clinical and scientific researchers to conduct related research in the future.

## Author contributions

**Conceptualization:** Hongshuo Shi, Min Peng, Guomin Si, Fengshan Sun.

**Data curation:** Hongshuo Shi, Chengda Dong, Wenbin Liu, Min Peng.

**Formal analysis:** Chengda Dong.

**Funding acquisition:** Min Peng, Guomin Si, Fengshan Sun.

**Methodology:** Chengda Dong, Min Peng.

**Software:** Chengda Dong, Wenbin Liu, Min Peng.

**Writing – original draft:** Hongshuo Shi, Wenbin Liu.
